# Landscape of *KRAS*^G12C^, Associated Genomic Alterations, and Interrelation With Immuno-Oncology Biomarkers in *KRAS*-Mutated Cancers

**DOI:** 10.1200/PO.21.00245

**Published:** 2022-03-23

**Authors:** Mohamed E. Salem, Sherif M. El-Refai, Wei Sha, Alberto Puccini, Axel Grothey, Thomas J. George, Jimmy J. Hwang, Bert O'Neil, Alexander S. Barrett, Kunal C. Kadakia, Laura W. Musselwhite, Derek Raghavan, Eric Van Cutsem, Josep Tabernero, Jeanne Tie

**Affiliations:** ^1^ Levine Cancer Institute, Atrium Health, Charlotte, NC; ^2^ Tempus Labs Inc, Chicago, IL; ^3^ University of Genoa, Ospedale Policlinico San Martino IRCCS, Genoa, Italy; ^4^ West Cancer Center, Germantown, TN; ^5^ University of Florida, Gainesville, FL; ^6^ University Hospitals Gasthuisberg, Leuven & KULeuven, Leuven, Belgium; ^7^ Vall d’Hebron Hospital Campus and Institute of Oncology (VHIO), IOB-Quiron, UVic-UCC, Barcelona, Spain; ^8^ Peter MacCallum Cancer Centre, Melbourne, Australia; ^9^ Walter + Eliza Hall Institute of Medical Research, Melbourne, Australia

## Abstract

Promising single-agent activity from sotorasib and adagrasib in *KRAS*^G12C^-mutant tumors has provided clinical evidence of effective KRAS signaling inhibition. However, comprehensive analysis of *KRAS*-variant prevalence, genomic alterations, and the relationship between *KRAS* and immuno-oncology biomarkers is lacking.

**MATERIALS AND METHODS:**

Retrospective analysis of deidentified records from 79,004 patients with various cancers who underwent next-generation sequencing was performed. Fisher's exact test evaluated the association between cancer subtypes and *KRAS* variants. Logistic regression assessed *KRAS*^G12C^ comutations with other oncogenes and the association between *KRAS* variants and immuno-oncology biomarkers.

**RESULTS:**

Of the 79,004 samples assessed, 13,758 (17.4%) harbored *KRAS* mutations, with 1,632 (11.9%) harboring *KRAS*^G12C^ and 12,126 (88.1%) harboring other *KRAS* variants (*KRAS*^non-G12C^). Compared with *KRAS*^non-G12C^ across all tumor subtypes, *KRAS*^G12C^ was more prevalent in females (56% *v* 51%, false discovery rate-adjusted *P* value [FDR-*P*] = .0006), current or prior smokers (85% *v* 56%, FDR-*P* < .0001), and patients age > 60 years (73% *v* 63%, FDR-*P* ≤ .0001). The most frequent *KRAS* variants across all subtypes were G12D (29.5%), G12V (23.0%), G12C (11.9%), G13D (6.5%), and G12R (6.2%). *KRAS*^G12C^ was most prevalent in patients with non–small-cell lung cancer (9%), appendiceal (3.9%), colorectal (3.2%), tumor of unknown origin (1.6%), small bowel (1.43%), and pancreatic (1.3%) cancers. Compared with *KRAS*^non-G12C^-mutated, *KRAS*^G12C^-mutated tumors were significantly associated with tumor mutational burden-high status (17.9% *v* 8.4%, odds ratio [OR] = 2.38; FDR-*P* < .0001). *KRAS*^G12C^-mutated tumors exhibited a distinct comutation profile from *KRAS*^non-G12C^-mutated tumors, including higher comutations of *STK11* (20.59% *v* 5.95%, OR = 4.10; FDR-*P* < .01) and *KEAP1* (15.38% *v* 4.61%, OR = 3.76; FDR-*P* < .01).

**CONCLUSION:**

This study presents the first large-scale, pan-cancer genomic characterization of *KRAS*^G12C^. The *KRAS*^G12C^ mutation was more prevalent in females and older patients and appeared to be associated with smoking status. *KRAS*^G12C^ tumors exhibited a distinct comutation profile and were associated with tumor mutational burden-high status.

## INTRODUCTION

Kirsten rat sarcoma viral oncogene homolog (*KRAS*) is the most common driver oncogene in human cancers; it is an essential mediator of tumor cell growth and survival^1^ and is often associated with poor outcomes.^2,3^ More than three decades of efforts to target KRAS downstream signaling pathways in *KRAS*-mutated cancers have largely been ineffective.^4^

**CONTEXT**

**Key Objective**
What is the prevalence of *KRAS*^G12C^ and associated genomic alterations, and what is the relationship between *KRAS*^G12C^ mutation status and immune-related biomarkers?
**Knowledge Generated**
Among 79,004 patients, *KRAS*^G12C^ was more often identified in females, current or prior smokers, and older patients. *KRAS*^G12C^ was most prevalent in patients with non–small-cell lung cancer, appendiceal, colorectal, tumor of unknown origin, small bowel, and pancreatic cancers. *KRAS*^G12C^ tumors were genomically distinct from *KRAS*^non-G12C^ tumors, including variations in *STK11*, *KEAP1*, and other gene frequencies. TMB-high was strongly associated with tumors harboring *KRAS*^G12C^.
**Relevance**
*KRAS*^G12C^ tumors exhibited a distinct comutation profile from *KRAS*^non-G12C^ tumors. Additionally, tumor mutational burden-high status was associated with *KRAS*^G12C^ tumors, suggesting a potential role for combination strategies with immunotherapy. These results may guide future therapeutic strategies.

The recent development of KRAS^G12C^-selective inhibitors of GTP binding—locking KRAS in an inactive state^5,6^—established a foundation for the development of inhibitors suitable for clinical testing and reignited interest in this historically undruggable target. Promising single-agent activity from AMG510 (sotorasib) and MRTX849 (adagrasib) in *KRAS*^*G*12C^-mutant tumors, especially in non–small-cell lung cancer (NSCLC) and colorectal cancer (CRC), has provided the first clinical evidence of *KRAS*-mutant tumor inhibition^7-9^ and led to the subsequent US Food and Drug Administration breakthrough therapy designation for sotorasib in locally advanced or metastatic NSCLC. Four other KRAS^G12C^ inhibitors (JNJ-74699157, JDQ443, GDC-6036, and LY3499446) have now entered the clinic.

Beyond single-gene alterations such as *KRAS*^G12C^, there are various broad genomic biomarkers associated with treatment response or resistance in patients with cancer, many of which are interrelated. Recent evidence suggests clinically relevant interactions between *RAS* mutations and immuno-oncology (IO) biomarkers.^10,11^ However, the extent of relationships between microsatellite instability-high (MSI-H)/mismatch repair deficient status, tumor mutational burden (TMB), programmed death ligand 1 (PD-L1), and *KRAS* mutations remains unclear.

Here, we analyzed next-generation sequencing (NGS) data from patients with various cancer subtypes to characterize the prevalence of *KRAS*^G12C^ and other *KRAS* variants, identify associated genomic alterations, and describe the relationship between *KRAS* mutation status and IO biomarkers, which may provide guidance for future therapeutic strategies.

## MATERIALS AND METHODS

### Clinical Data

Clinical data were extracted from the Tempus Labs (Chicago, IL) real-world oncology database, as previously described^12^ (Data Supplement).

### NGS Profiling of Tumor Samples

Tumor samples were clinically profiled at a College of American Pathologists-accredited, Clinical Laboratory Improvement Amendments-certified laboratory (Tempus Labs, Chicago, IL) using a single platform. Tissue-based NGS with the Tempus xT laboratory developed test was performed on DNA and RNA isolated from formalin-fixed, paraffin-embedded tumor samples using the NovaSeq and HiSeq platforms (Illumina, San Diego, CA), similar to previously described methods^12,13^ (Data Supplement).

### IO Markers

Records included for assessment of IO biomarkers were restricted to baseline tissue samples profiled with the Tempus xT assay. TMB, MSI, and PD-L1 expression analyses are reported in the Data Supplement.^14,15^

### Statistical Analysis

Chi-square tests were used to evaluate associations between *KRAS*^G12C^ status and patient demographic variables. Logistic regression analyzed associations between cancer subtypes and *KRAS* variants, associations between *KRAS* variants and IO biomarkers, and comutations between *KRAS* ^G12C^ and other oncogenes. To control false discovery rate (FDR) because of multiple testing, the Benjamini-Hochberg procedure^16^ was used to calculate FDR-adjusted *P* value (FDR-*P*), with FDR-*P* < .05 considered statistically significant. All analyses were completed with SAS 9.4 (SAS Institute Inc, Cary, NC).

### Ethics Statement

All data were deidentified in accordance with the Health Insurance Portability and Accountability Act before investigation and granted institutional review board oversight exemption (Pro00042950).

## RESULTS

### Patient and Tumor Characteristics

From 79,004 tumor samples analyzed, 13,758 *KRAS*-mutated tumors were identified from various cancer types (Data Supplement). Of these, 1,632 (11.9%) tumors harbored the *KRAS*^G12C^ variant while 12,126 harbored other *KRAS* variants (*KRAS*^non-G12C^; Table 1 and Data Supplement). An overview of demographics and clinical characteristics stratified by *KRAS*^G12C^ mutation status is summarized in Table 1. When compared with *KRAS*^non-G12C^ across all tumor subtypes, *KRAS*^G12C^ was more often identified in females (56% *v* 51%; FDR-*P* = .0006), current or prior smokers (85% *v* 56%; FDR-*P* < .0001), and patients older than 60 years (73% *v* 63%; FDR-*P* < .0001).

**TABLE 1.**
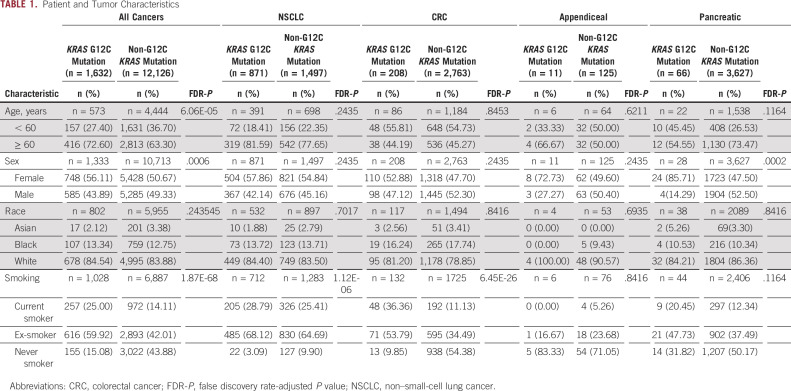
Patient and Tumor Characteristics

### *KRAS*-Variant Distribution

**FIG 1.**
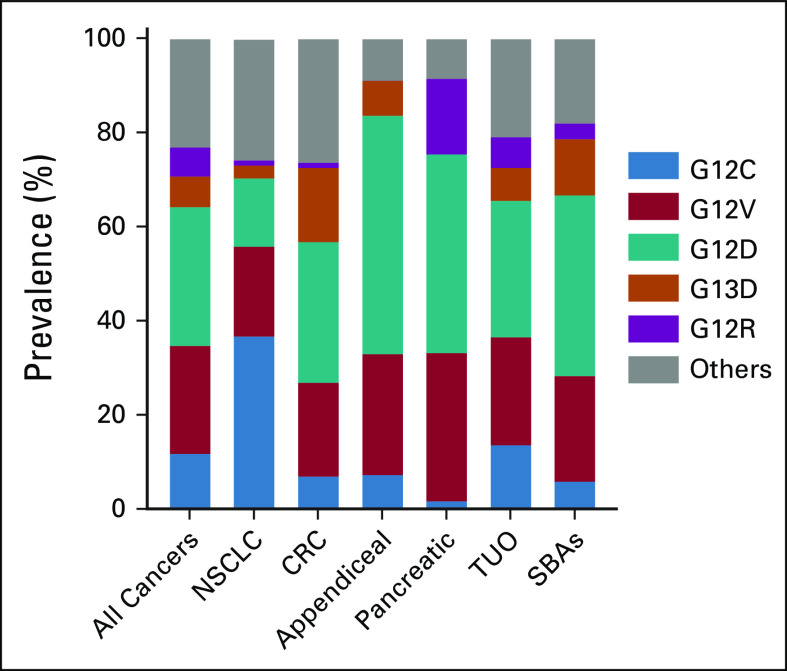
Prevalence of *KRAS* variants by tumor subtype. Most common *KRAS* mutation variants observed in all *KRAS*-mutated tumors (n = 13,758) and subtypes. CRC, colorectal cancer; NSCLC, non–small-cell lung cancer; SBA, small bowel adenocarcinoma; TUO, tumor of unknown origin.

The most frequent *KRAS* variants across all tumors analyzed were G12D (29.5%), G12V (23.0%), G12C (11.9%), G13D (6.5%), and G12R (6.2%). However, the distribution of *KRAS* variants significantly differed by cancer type (Fig 1 and Data Supplement).

**FIG 2.**
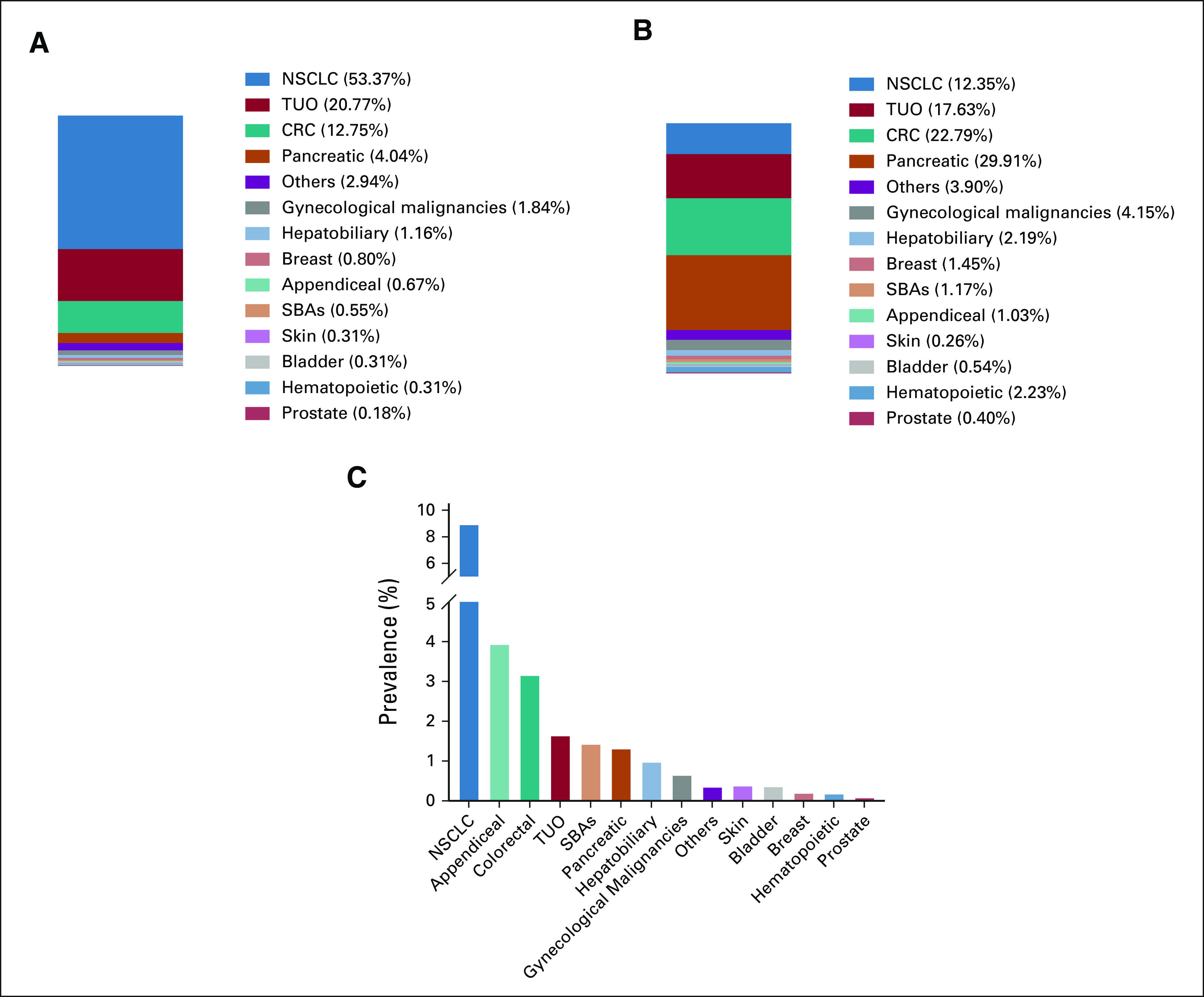
Frequency of *KRAS*^G12C^ and *KRAS*^non-G12C^ by cancer subtypes. (A) Distribution of 1,632 patients with confirmed *KRAS*^G12C^ by tumor subtype. (B) Distribution of 12,126 patients with other confirmed *KRAS* variants (*KRAS*^non-G12C^) by tumor subtype. Cancer subtype distribution was significantly different between G12C and non-G12C *KRAS* mutation groups (*P* < .0001). (C) Frequency of *KRAS*^G12C^ mutations in 14 cancer types. CRC, colorectal cancer; NSCLC, non–small-cell lung cancer; SBA, small bowel adenocarcinoma; TUO, tumor of unknown origin.

Within the *KRAS*^G12C^-mutated cohort, NSCLC had the highest prevalence (53%), followed by tumors of unknown origin (TUO; 21%) and CRC (13%; Fig 2A). In contrast, within the *KRAS*^non-G12C^ cohort, pancreatic tumors had the highest *KRAS* mutation prevalence (30%), followed by CRC (23%) and TUO (18%; Fig 2B). A complete list of pan-cancer *KRAS*-variant distribution is presented in the Data Supplement.

### *KRAS*^G12C^ Distribution

Cancer subtype distribution was significantly different between *KRAS*^G12C^ and *KRAS*^non-G12C^ mutation groups (P < .0001; Fig 2C). *KRAS*^G12C^ was most prevalent in patients with NSCLC (8.9%), appendiceal cancer (3.9%), CRC (3.2%), TUO (1.6%), small bowel adenocarcinomas (1.4%), and pancreatic cancer (1.3%). Hepatobiliary, hematopoietic, breast, bladder, prostate, and skin cancers had a *KRAS*^G12C^ frequency rate of < 1% each.

In NSCLC, *KRAS*^G12C^ was mostly associated with adenocarcinoma histology compared with squamous cell carcinoma (92.4% *v* 3.4%, *P* < .0001). Smoking status was also associated with the presence of G12C among patients with NSCLC harboring *KRAS* variants (FDR-*P* < .0001). In CRC, no differences were observed based on age, sex, or race when comparing the *KRAS*^G12C^ versus *KRAS*^non-G12C^ populations, but smoking status was associated with *KRAS*^G12C^ (FDR-*P* < .0001).

### Association Between *KRAS*^G12C^ Mutations and Mutations in Other Oncogenes

**FIG 3.**
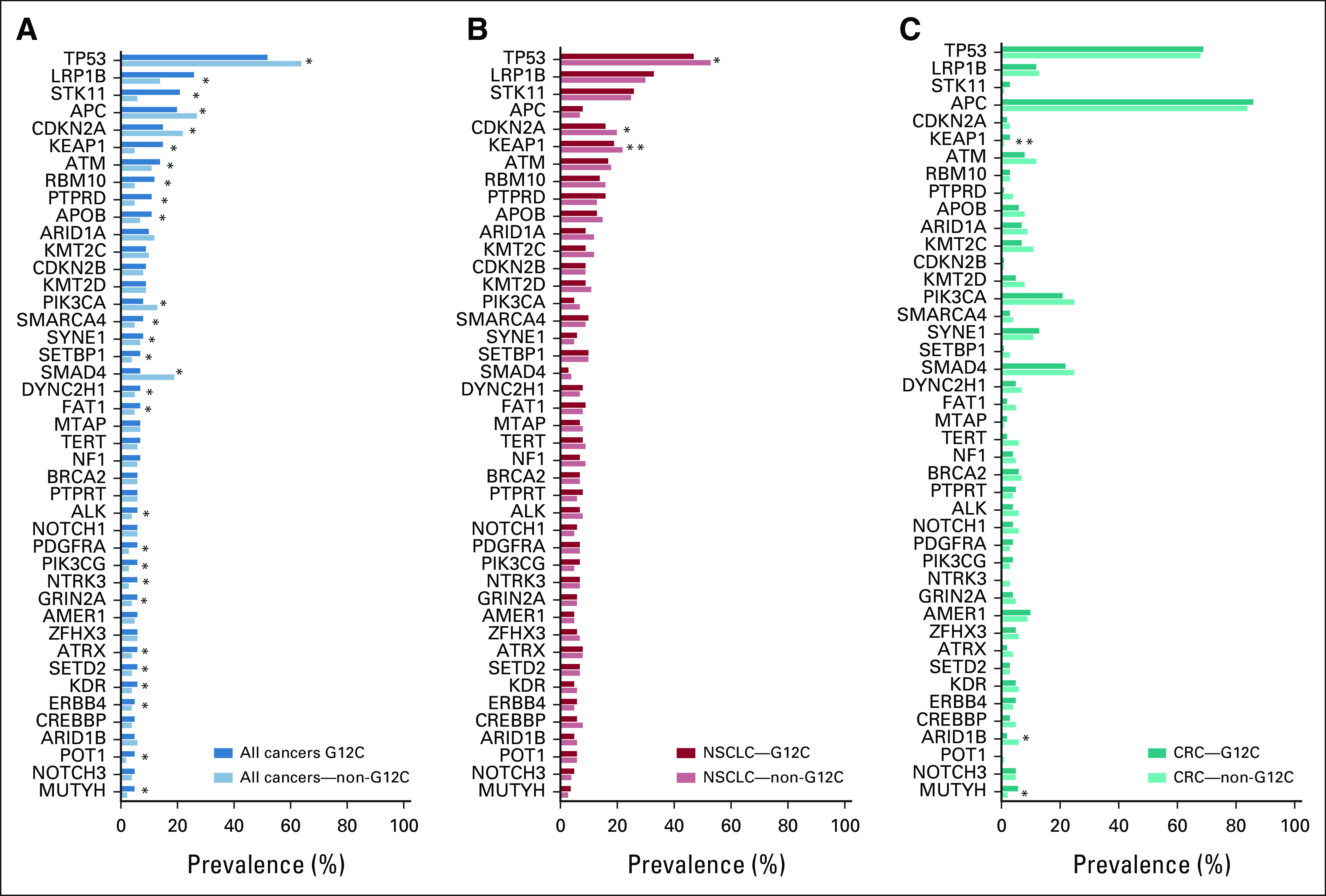
Comparison of oncogenic comutations for *KRAS*^G12C^ and *KRAS*^non-G12C^ cohorts. Comparison of comutations identified in the *KRAS*^G12C^- and *KRAS*^non-G12C^-mutated cohorts. Comutations altered in more than 5% of patients with a confirmed *KRAS* mutation were included and are shown by subgroups: (A) all cancers, (B) NSCLC, and (C) CRC. Logistic regression was used to calculate the OR and 95% CIs (Data Supplement). FDR-*P* < .05 was considered statistically significant. Some comutations with *KRAS*^G12C^ in all cancers could be caused by coenrichment of the oncogene mutation and *KRAS*^G12C^ mutation in NSCLC, as described in the Results section. Careful interpretation of the analysis results using the Data Supplement is recommended. *Significant G12C status (*KRAS*^G12C^ and *KRAS*^non-G12C^) effect on the mutation of the oncogene in the subgroup (all cancers or NSCLC or CRC) at FDR-*P* < .05. **Significant G12C status (*KRAS*^G12C^ and *KRAS*^non-G12C^) × cancer subtype (NSCLC or CRC) interaction effect on the mutation of the oncogene at FDR-*P* < .05. CRC, colorectal cancer; FDR-*P*, false discovery rate-adjusted *P* value; NSCLC, non–small-cell lung cancer; OR, odds ratio.

Among patients with a confirmed *KRAS* mutation, tumors harboring *KRAS*^G12C^ exhibited a distinct comutation profile compared with those with *KRAS*^non-G12C^ mutations (Fig 3).

For instance, *STK11* (20.59% *v* 5.95%, odds ratio [OR] = 4.10), *KEAP1* (15.38% *v* 4.61%, OR = 3.76), and *MUTYH* (4.96% *v* 2.35%, OR = 2.17) were more frequently mutated in *KRAS*^G12C^-mutant tumors across all cancer types (*P* < .001 and FDR-*P* < .01 for all comparisons). Meanwhile, *SMAD4* (7.23% *v* 19.05%, OR = 0.33), *TP53* (52.39% *v* 64.25%, OR = 0.61), *CDKN2A* (15.44% *v* 22.37%, OR = 0.63), and *PIK3CA* (8.03% *v* 12.59%, OR = 0.61) were less frequently mutated in *KRAS*^G12C^ compared with *KRAS*^non-G12C^-mutant tumors (*P* < .0001 and FDR-*P* < .0001 for all comparisons). Notably, although none of the tumors harboring *KRAS*^G12C^ exhibited *BRAF*^V600E^ comutations, *BRAF*^non-V600E^ comutations were observed in 3.1% of *KRAS*^G12C^-mutant tumors. Since some other mutations (eg, *STK11* and *KEAP1*) are enriched in NSCLC and the *KRAS*^G12C^ mutation is also enriched in NSCLC, the association between these genes and *KRAS*^G12C^ may be caused by the coenrichment in NSCLC rather than a true association across all tumors. Therefore, to rule out comutation because of coenrichment in NSCLC, we further stratified the comutation analysis according to patients with and without NSCLC (Data Supplement).

In CRC, *MUTYH* (5.77% *v* 2.28%, OR = 2.62, *P* = .005 and FDR*-P* = .027) and *ARID1B* (1.92% *v* 6.26%, OR = 0.29, *P* = .009, and FDR*-P* = .042) mutational frequencies were significantly different between tumors with *KRAS*^G12C^ versus *KRAS*^non-G12C^ mutations.

Several genes were found to have significant subtype effects, meaning prevalence of the oncogene mutation was significantly different across cancer subtypes. However, the *KRAS*^G12C^ effect was not significantly different between NSCLC and CRC for most oncogenes evaluated, with only *KEAP1* exhibiting different comutation patterns between NSCLC and CRC (FDR-*P* < .05). A full list of comutations by cancer type is presented in the Data Supplement.

### Association Between *KRAS*-Mutated Versus *KRAS* Wild-Type Tumors and IO Biomarkers

We examined the association between *KRAS* mutation status and IO biomarkers (Data Supplement). Compared with *KRAS*-wild-type (WT) tumors, TMB-high status was less frequent in *KRAS*-mutated tumors both in the overall cohort (9.48% *v* 10.51%, OR = 0.89, and FDR-*P* = .004) and in the CRC cohort (4.68 *v* 10.34%, OR = 0.43, and FDR-*P* < .0001). This association was not observed when considering only NSCLC tumors. A similar association was observed between MSI-H and *KRAS* mutational status both in the overall cohort and CRC. Conversely, in NSCLC, high PD-L1 expression was more frequently observed in *KRAS*-mutated tumors compared with *KRAS*-WT (65.3% *v* 58.5%, OR = 1.34, and FDR-*P* = .0002).

### The Association Between *KRAS*^G12C^, *KRAS*^non-G12C^, *KRAS* WT, and IO Biomarkers

We further stratified the IO biomarker analysis by separating *KRAS*^G12C^-mutated tumors from those harboring *KRAS*^non-G12C^ mutations. The relationships between *KRAS* mutational status (*KRAS*^G12C^ mutations, *KRAS*^non-G12C^ mutations, and *KRAS* WT) and IO biomarkers are reported in the Data Supplement. Overall, the frequency of TMB-high status was found to be significantly different between the three *KRAS* mutation groups (FDR-P < .0001). TMB-high status was more frequently associated with *KRAS*^G12C^ compared with *KRAS*-WT (17.9% *v* 10.51%, OR = 1.86) and *KRAS*^non-G12C^ mutations (17.9% *v* 8.4%, OR = 2.38) and less frequent in *KRAS*^non-G12C^–mutated compared with *KRAS*-WT tumors (8.40% *v* 10.51%, OR = 0.78). In CRC, TMB-high status was less frequent in both *KRAS*^G12C^- and *KRAS*^non-G12C^-mutated tumors compared with *KRAS*-WT tumors (4.40% and 4.70% *v* 10.34%, OR = 0.3992 and 0.4271, and FDR-*P* < .0001). The association between TMB-high and *KRAS* mutation status was not significant when considering only NSCLC (Data Supplement).

A significant association was observed between PD-L1 expression levels and the three *KRAS* groups (FDR-*P* < .0001) when including all cancer types. High expression was more frequently associated with *KRAS*^G12C^ compared with *KRAS*-WT (53.96% *v* 41.50%, OR = 1.65) and *KRAS*^non-G12C^-mutant tumors (53.96% *v* 40.40%, OR = 1.73) and less frequent in *KRAS*^non-G12C^ compared with *KRAS*-WT tumors (40.40% *v* 41.50%, OR = 0.96). However, in a stratified analysis separately considering patients with and without NSCLC, the high expression of PD-L1 was not significantly different between *KRAS*^G12C^ and *KRAS*^non-G12C^ mutations in either NSCLC (OR = 1.16, FDR-*P* = .38) or non-NSCLC tumors (OR = 1.01, FDR-*P* = .95), suggesting that the significant association between PD-L1 and *KRAS*^G12C^ observed in all cancers was likely due to coenrichment in NSCLC.

**FIG 4.**
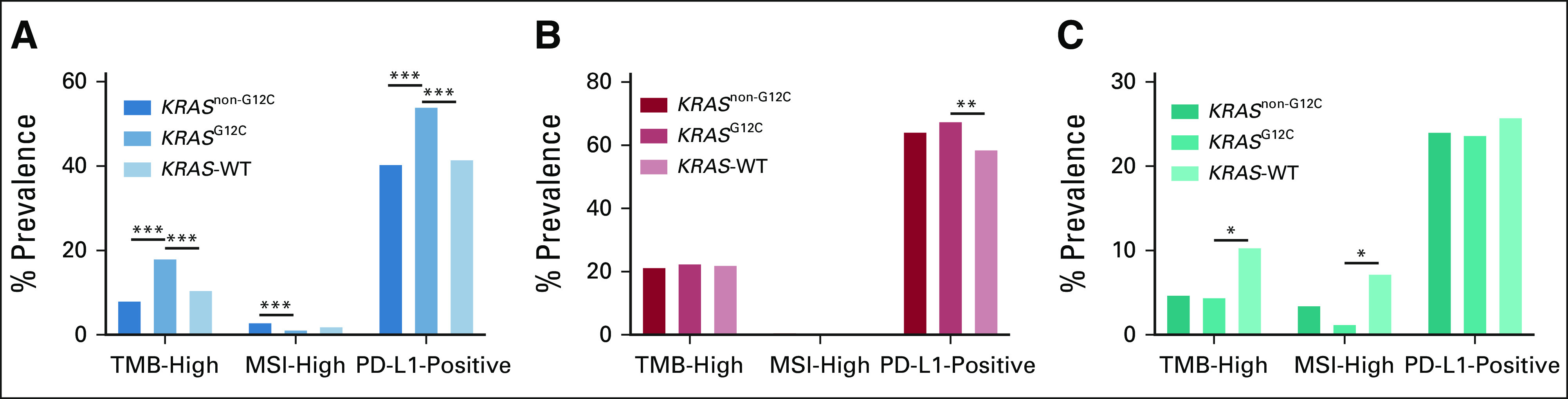
Evaluation of immune biomarkers by *KRAS*^G12C^, *KRAS*^non-G12C^, and *KRAS* WT. Comparison of TMB-high (defined as > 10 mut/Mb), high PD-L1 expression, and MSI-high cases across three *KRAS* cohorts (*KRAS*^G12C^, *KRAS*^non-G12C^, and *KRAS*-WT). The association between PD-L1 and *KRAS*^G12C^ in all cancers could be caused by coenrichment of PD-L1–positive and *KRAS*^G12C^ mutation in NSCLC, as described in the Results section: (A) all cancers, (B) NSCLC, and (C) CRC. FDR-*P* < .05 was considered statistically significant. ***FDR-*P* < .0001; **FDR-*P* ≥ .0001 and FDR-*P* < .01; *FDR-*P* ≥ .01 and FDR-*P* < .05. CRC, colorectal cancer; FDR-*P*, false discovery rate-adjusted *P* value; MSI, microsatellite instability; mut/Mb, mutations per megabase; NSCLC, non–small-cell lung cancer; PD-L1, programmed death ligand 1; TMB, tumor mutational burden; WT, wild-type.

In NSCLC, high PD-L1 expression was more frequent in both *KRAS*^G12C^- and *KRAS*^non-G12C^-mutated tumors compared with *KRAS*-WT tumors (67.53% and 64.17% *v* 58.54%, OR = 1.47 and 1.27, and FDR-*P* = .0017 and .0123). A nonsignificant association between high PD-L1 expression and *KRAS* mutation status was seen in CRC (Fig 4).

Finally, MSI-H status was significantly different across the three *KRAS* groups in the overall population (FDR-*P* < .0001). MSI-H was less frequently associated with *KRAS*^G12C^ compared with *KRAS*-WT (1.17% *v* 1.92%, OR = 0.63) and *KRAS*^non-G12C^-mutated tumors (1.17% *v* 2.86%, OR = 0.39) and more frequently associated with *KRAS*^non-G12C^-mutated when compared with *KRAS*-WT tumors (2.86% *v* 1.92%, OR = 1.59; Data Supplement).

## DISCUSSION

Despite being the most frequently mutated oncogene in human cancers, therapeutic targeting of *KRAS*-driven tumors remains a formidable challenge. Several promising KRAS^G12C^ inhibitors have now entered the clinic and combination strategies with chemotherapy, immune checkpoint inhibitors, anti-epidermal growth factor receptor (EGFR) antibodies, and pan-KRAS targeting agents (eg, SOS1 and SHP2 inhibitors) are being explored—holding the potential for transforming clinical management of *KRAS*-mutated solid tumors. Hence, understanding variations in *KRAS* mutational frequencies, clinicopathological characteristics, and comutations for *KRAS*^G12C^ tumors across cancer types, as well as their interplay with predictors of response to immune checkpoint inhibitors, may advance the clinical development of KRAS^G12C^ inhibitors.

In the current study, we observed distinct patterns in *KRAS* mutations where the position and type of substitution varied between different cancers. *KRAS*^G12C^ was most frequent in NSCLC and TUOs, suggesting that a large proportion of these TUOs may have originated from the lung. The prevalence within individual cancer subtypes observed in our study was similar to findings from the The Cancer Genome Atlas data set (NCI GDC data portal, v29.0)^16^ and a recently published study.^17^

The association between cancer type and *KRAS* mutational status can be partially explained by tissue-specific differential exposure to mutagens such as tobacco smoke. For example, in lung cancer, the G:C → T:A transversion causing the G12C mutation is predominantly seen in smokers while never smokers are more likely to have a transition mutation (G:C→A:T).^18,19^ In NSCLC tumors, the *KRAS*^G12C^ variant was almost exclusively detected in tissue from current or former smokers. More intriguing, however, was the striking enrichment of *KRAS*^G12C^ mutations in patients with CRC with a smoking history, which to our knowledge has not been previously reported. Among all CRC cases with *KRAS*^G12C^ mutations, 90% were in current or former smokers while only 46% of *KRAS*^non-G12C^ mutations were associated with smoking. There was a similar trend in pancreatic cancer (68% *v* 50%), but the power of this observation is limited by the small number of *KRAS*^G12C^-mutant cases. Similar to lung cancer, cigarette smoking is linked to an increased risk for CRC albeit with a much more modest association, which likely reflects the tissue-specific differences in exposure to individual tobacco mutagens and the lower prevalence of *KRAS*^G12C^ mutations in CRC.^20,21^ Accumulating evidence indicates that the smoking-related risk in CRC may be limited to molecularly defined subsets, with several studies reporting that smoking is associated with increased risk of *BRAF*-mutated and MSI-H CRC but not *BRAF*-WT, MSS, or *KRAS*-mutant tumors.^22-26^ Previous studies^26-28^ have not found an overall association between smoking variables and CRC tumors with transversion or transition mutations in *KRAS*; however, these studies are limited by the small number of patients who had identified *KRAS* mutations, challenges with the accuracy of tobacco use documented in medical records, and lack of post hoc analyses correlating smoking status with discrete *KRAS* point mutations. Given that *KRAS* mutations are all not created equal, where various mutations have been shown to impart unique biochemical effect and different oncogenic signaling, it is plausible that the causal link to tobacco may be limited to certain *KRAS* mutations.

Direct KRAS^G12C^ inhibitors undoubtedly represent a major leap forward for the treatment of *KRAS*-mutant cancers; however, a predicted challenge to their clinical development is the high degree of biological heterogeneity in these tumors, which is likely to affect therapeutic response to KRAS inhibition. In NSCLC, objective response rates from early-phase studies with KRAS^G12C^ inhibitors were lower than agents targeting *EGFR*-activating mutations or *ALK*-*RET* fusions.^29,30^ This suggests greater biological diversity and oncogenic pathway redundancy in *KRAS*^G12C^-mutant tumors compared with tumors driven by other oncogenes. Furthermore, co-occurrence of *KRAS* mutations with other oncogenes such as *TP53* and *CDKN2A* in various cancers, *KEAP1* and *STK11* in lung adenocarcinoma, or *APC* and *PIK3CA* in CRC, may influence therapeutic response.^31,32^ Overall, we found significantly higher comutation rates between *KRAS*^G12C^ and several other oncogenes compared with *KRAS*^non-G12C^-mutant tumors, although these differences were not observed in the NSCLC and CRC cohorts. Specifically, we observed similar comutation patterns between *KRAS*^G12C^- and *KRAS*^non-G12C^-mutant NSCLC for key oncogenes *LRP1B*, *STK11*, *KEAP1*, and *CDKN2A* but a lower comutation rate with *TP53*. Lung cancer cells with *KRAS/LRP1B* comutation have been reported to be sensitive to HSP90 inhibitors,^31^ and tumors with *KRAS/TP53* comutation have demonstrated increased PD-L1 expression and a remarkable clinical benefit to pembrolizumab.^33^ Additionally, preclinical work with adagrasib suggests that *KRAS*^G12C^- /*STK11*-mutated NSCLC could be targeted with a combination of KRAS^G12C^ inhibition and an RTK or mTOR inhibitor, whereas *KRAS*^G12C^- /*CDKN2A*-mutated NSCLC could be more effectively treated by combination with a CDK4/6 inhibitor.^34^ Collectively, this suggests that the role of comutation should be considered in clinical trials targeting *KRAS*^G12C^-mutant tumors.

In the phase I clinical trial, the overall response rate in CRC with sotorasib alone was limited at 7.1%. The results of progression-free survival in CRC were also worse than those observed in NSCLC.^35^ Consistent with this, Amodio et al^36^ observed that KRAS^G12C^ inhibitors produce less profound and more transient inhibition of KRAS downstream signaling in CRC compared with NSCLC models. Akin to targeting *BRAF*^*V600E*^ CRC with BRAF inhibitors, EGFR signaling rebound was also found to be the dominant mechanism of CRC resistance to KRAS^G12C^ inhibition. Of clinical relevance, targeting both EGFR and KRAS^G12C^ was highly effective in preclinical CRC models. This combinatorial approach is currently being explored in several phase I-III trials (NCT04793958, NCT04449874, NCT03785249, and NCT04185883). As with most targeted treatments, primary or acquired resistance to these combination therapies will be the rule rather the exception. Understanding comutation patterns in *KRAS*^G12C^-mutant CRC may shed light on further therapeutic strategies. Here, similar to other reports,^37,38^ we observed a statistically significant association between *KRAS*^G12C^ and *MUTYH* (5.8% *v* 2.3%, OR: 2.62 [1.26-5.01]) and between *KRAS*^G12C^ and *ARID1B* (1.9% *v* 6.3%, OR: 0.29 [0.08-0.78], compared with *KRAS*^non-G12C^-mutant CRC. However, we did not observe a lower comutation rate for *NOTCH3* or *PIK3CA* in *KRAS*^G12C^-mutated CRC, as reported recently by Henry et al.^39^ In our cohort, *PIK3CA* mutations were found in 21% of *KRAS*^G12C^-mutated CRC. Cotargeting PI3K and MEK/ERK signaling in *KRAS*-mutant tumors has emerged as a promising therapeutic strategy in preclinical studies, but successful clinical development of this combination has been hampered by dose-limiting toxicities.^40^

There has been substantial interest in the interaction between specific molecular changes and the immune system. Despite the strong association with smoking, we did not observe a difference in the frequency of TMB-high tumors between *KRAS*^G12C^-mutant, *KRAS*^non-G12C^-mutant, and *KRAS*-WT NSCLC; in fact, we found a surprisingly lower frequency of TMB-high status in *KRAS*^G12C^-mutant compared with *KRAS*-WT CRC. Overall, *KRAS*^G12C^ tumors had higher frequencies of PD-L1 positivity than *KRAS*^non-G12C^-mutant and *KRAS*-WT tumors. In NSCLC, the higher frequency was maintained when comparing *KRAS*^G12C^ and *KRAS*-WT tumors. Arbor et al recently reported a higher median PD-L1 expression in *KRAS*^G12C^- vs. *KRAS*^non-G12C^-mutated NSCLC, but similar to our study, the proportion of patients with PD-L1–positive expression (TPS ≥ 1%) was similar in *KRAS*^G12C^- and *KRAS*^non-G12C^-mutated patients.^18^ Preclinical models demonstrated that treating *KRAS*^G12C^-mutant tumors with sotorasib resulted in proinflammatory tumor microenvironments that were highly responsive to immune checkpoint inhibition (ICI),^41^ underpinning the rationale for ongoing trials combining KRAS^G12C^ inhibitors and ICI (NCT04613596, NCT03785249, NCT04185883, and NCT04449874). Whether dual KRAS^G12C^ and ICI will be more efficacious than ICI alone in PD-L1–positive *KRAS*^G12C^-mutant NSCLC remains to be seen.

Treatment and outcome data were not available for the entirety of this data set, limiting the correlation between molecular subsets and treatment-specific outcomes. There was also missing information on patient age and race, which may limit the power to detect any statistical difference in these variables between *KRAS*^G12C^- and *KRAS*^non-G12C^–mutated cancers. We used a TMB cutoff of 10 mut/Mb to define TMB-high versus TMB-low across all tumor types, but the optimal threshold to identify a cancer as TMB-high for ICI treatment selection remains the subject of much debate.

In conclusion, to our knowledge, this is the first comprehensive analysis of *KRAS*^G12C^ distribution, associated comutations, PD-L1 expression levels, and TMB in a pan-cancer data set. *KRAS*^G12C^ mutation rates varied widely among different cancer types. Tumor mutational burden-high was strongly associated with tumors harboring *KRAS*^G12C^, which could potentially help identify additional responders to ICI plus KRAS^G12C^ inhibitor combination treatment. These findings provide baseline data for the prevalence of molecular and histologic parameters potentially associated with responsiveness to KRAS^G12C^ inhibitors in several malignancies.

## Data Availability

A data sharing statement provided by the authors is available with this article at DOI https://doi.org/10.1200/PO.21.00245.
